# IgA-deficient humans exhibit gut microbiota dysbiosis despite secretion of compensatory IgM

**DOI:** 10.1038/s41598-019-49923-2

**Published:** 2019-09-19

**Authors:** Jason R. Catanzaro, Juliet D. Strauss, Agata Bielecka, Anthony F. Porto, Francis M. Lobo, Andrea Urban, Whitman B. Schofield, Noah W. Palm

**Affiliations:** 1 Section of Pulmonology, Allergy, Immunology, and Sleep Medicine, Department of Pediatrics, Yale School of Medicine, New Haven, CT USA; 2 Department of Immunobiology, Yale School of Medicine, New Haven, CT USA; 3 Artizan Biosciences, New Haven, CT USA; 4 Section of Pediatric Gastroenterology, Department of Pediatrics, Yale School of Medicine, New Haven, CT USA; 5 Section of Rheumatology, Allergy and Clinical Immunology, Department of Internal Medicine, Yale School of Medicine, New Haven, CT USA; 6 Section of Pediatric Endocrinology, Department of Pediatrics, Yale School of Medicine, New Haven, CT USA

**Keywords:** Mucosal immunology, Primary immunodeficiency disorders

## Abstract

Immunoglobulin A is the dominant antibody isotype found in mucosal secretions and enforces host-microbiota symbiosis in mice, yet selective IgA-deficiency (sIgAd) in humans is often described as asymptomatic. Here, we determined the effects of IgA deficiency on human gut microbiota composition and evaluated the possibility that mucosal secretion of IgM can compensate for a lack of secretory IgA. We used 16S rRNA gene sequencing and bacterial cell sorting to evaluate gut microbiota composition and taxa-specific antibody coating of the gut microbiota in 15 sIgAd subjects and matched controls. Despite the secretion of compensatory IgM into the gut lumen, sIgAd subjects displayed an altered gut microbiota composition as compared to healthy controls. These alterations were characterized by a trend towards decreased overall microbial diversity as well as significant shifts in the relative abundances of specific microbial taxa. While secretory IgA in healthy controls targeted a defined subset of the microbiota via high-level coating, compensatory IgM in sIgAd subjects showed less specificity than IgA and bound a broader subset of the microbiota. We conclude that IgA plays a critical and non-redundant role in controlling gut microbiota composition in humans and that secretory IgA has evolved to maintain a diverse and stable gut microbial community.

## Introduction

Secretory immunoglobulin A is the dominant antibody isotype in mucosal secretions and plays an essential role in defense against pathogenic microorganisms^1^. However, even in the absence of infection, the body produces approximately four grams of IgA daily, more than all other antibody isotypes combined^2^. Much of this IgA is secreted into the intestinal lumen, where it binds to and ‘coats’ specific members of the gut microbiota—the trillions of bacteria that constitutively colonize the human intestinal tract^3^.

Secretory IgA plays a crucial role in shaping the composition and function of the gut microbiota in mice^4,5^. Various genetically-modified mouse models that display defective secretory antibody responses exhibit dramatic alterations in gut microbial composition, function, and epithelial cell gene expression despite the production and secretion of compensatory IgM^6–11^. However, the role of IgA in shaping the composition and function of the microbiota in humans remained largely unexplored until recently.

Selective IgA deficiency (sIgAd), defined as a serum IgA concentration of less than 7 mg/dl with normal levels of serum IgG and IgM in subjects greater than four years of age, is the most common primary immunodeficiency in humans. Its incidence varies by geographical region, ranging from 1:700 in individuals of European descent to 1:18,500 in Japan^12^. While IgA deficiency is often described as “asymptomatic,” sIgAd subjects exhibit increased incidences of infectious, allergic, and autoimmune disorders including inflammatory bowel disease^13,14^. Nonetheless, the lack of consistent intestinal pathologies in sIgAd subjects suggests that additional immunological defense mechanisms may compensate for the lack of IgA-mediated protection at mucosal surfaces.

Here, we directly examine the effect of IgA deficiency on the human gut microbiota by defining the composition and stability of the gut microbiota in sIgAd subjects and controls. We confirm previous findings that secretory IgM can partially compensate for the lack of IgA in sIgAd subjects^15^. Nonetheless, sIgAd subjects exhibit modest but consistent and significant alterations in gut microbiota composition. Furthermore, compensatory secreted IgM in IgA-deficient subjects displays distinct microbial binding patterns as compared to conventional secretory IgA. Together, these studies reveal a unique and non-redundant role for secretory IgA in shaping gut microbial community structure in humans.

## Results

### sIgAd subjects exhibit compensatory secretion of microbiota-targeted IgM

**Figure 1:**
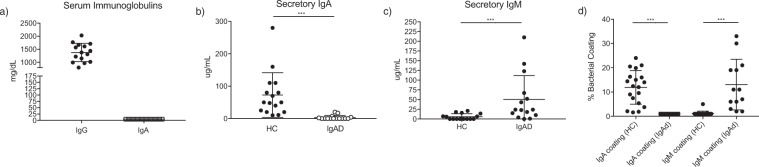
sIgAd subjects exhibit compensatory secretion of microbiota-targeted IgM. (**a**) Serum IgG and IgA levels for 15 sIgAd subjects. (**b**) Secretory IgA levels in the feces as measured by ELISA. (**c**) Secretory IgM levels in the feces as measured by ELISA. (**d**) Bacterial coating with IgA or IgM in the fecal microbiotas from fifteen healthy controls of sIgAd subjects. ***p < 0.001 via Mann-Whitney U test.

Fifteen subjects with reported sIgAd and fifteen age- and sex-matched healthy controls were recruited by mining electronic medical records and subjected to serum immunoglobulin analyses (Table 1). All subjects with reported IgA deficiency displayed serum IgA levels below the lower limit of detection at the time of study enrollment despite age-appropriate serum IgG titers (Fig. 1a). Six of the 15 sIgAd subjects had self-described increased incidences of upper respiratory infections compared to their peers. None of the IgA deficient subjects had received antibiotics in the 4 weeks prior to study participation. The average time between study participation and prior antibiotic usage was 6.5 months and six of 15 sIgAd subjects had no antibiotic usage in the year prior to enrollment (Table 2). One sIgAd subject had chronic diarrhea and nonspecific gastrointestinal discomfort without endoscopic evidence of Celiac disease or IBD, and three sIgAd subjects had a previously diagnosed autoimmune disease (two with type 1 diabetes mellitus and one with Celiac disease).

**Table 1:** Baseline characteristics of sIgAd and healthy subjects.

	sIgA deficient	HC	p value
Total Subjects	15	15	
Mean Age	31.7	29.7	0.66
Female Sex	11 (73%)	10 (67%)	0.99
Recurrent Infections	6 (40%)	0	
Autoimmune Disease	3 (20%)	0	
T1DM	2 (13%)	0	
Celiac Disease	1 (7%)	0	

Continuous and discrete variables were compared using the Mann-Whitney U test.

**Table 2:** sIgAd subjects, antibiotic usage, Chao1.

ID	Last Abx (months)	CHAO1
A1	1	100.3
A2	6	328.6
A7	>12	171
A8	1	130.8
A9	6	243.2
A10	>12	209.1
A11	1	140
A12	>12	264
A15	1	275.8
A16	>12	249.7
A17	1.5	175.1
A18	>12	178.6
A19	>12	147.8
A20	4	151.9
A21	3	179.9

sIgAd is normally diagnosed based on the absence of detectable IgA in the serum. Thus, we first examined whether subjects with serum IgA deficiency also lacked IgA in their stool. Subjects lacking serum IgA had on average 3.3 ± 1.7 μg/mL of secretory IgA in their stool compared to 72.8 ± 17.8 μg/mL in healthy controls (Fig. 1b). The polymeric Ig receptor, which is responsible for transporting IgA across the epithelium into the gut lumen, can also bind to and transport pentameric IgM. Thus, it has been suggested that IgM may at least partially compensate for the absence of IgA in subjects with sIgAd^16^. In line with this prediction, we found that sIgAd subjects displayed significantly higher levels of IgM in their stool than healthy controls (mean 50.4 ± 15.8 μg/mL in sIgAd versus 5.9 ± 1.9 ug/mL in controls) (Fig. 1c). The potential effect of secretory antibodies on the microbiota can also be measured by flow cytometric analysis of IgA or IgM coating of the gut microbiota (Supplemental Fig. 1)^3^. sIgAd subjects displayed undetectable IgA coating by flow cytometry while a significant fraction of bacteria in healthy controls was measurably coated with IgA (1% ± 0.2 versus 11.9% ± 1.8) (Fig. 1d). In contrast, sIgAd subjects showed significant bacterial coating with IgM (11.7% ± 2.7), which essentially mirrored the level of IgA coating seen in controls; in contrast, healthy controls exhibited minimal coating with IgM (1.3% ± 0.3) (Fig. 1d).

### sIgAd subjects harbor altered gut microbial communities

**Figure 2:**
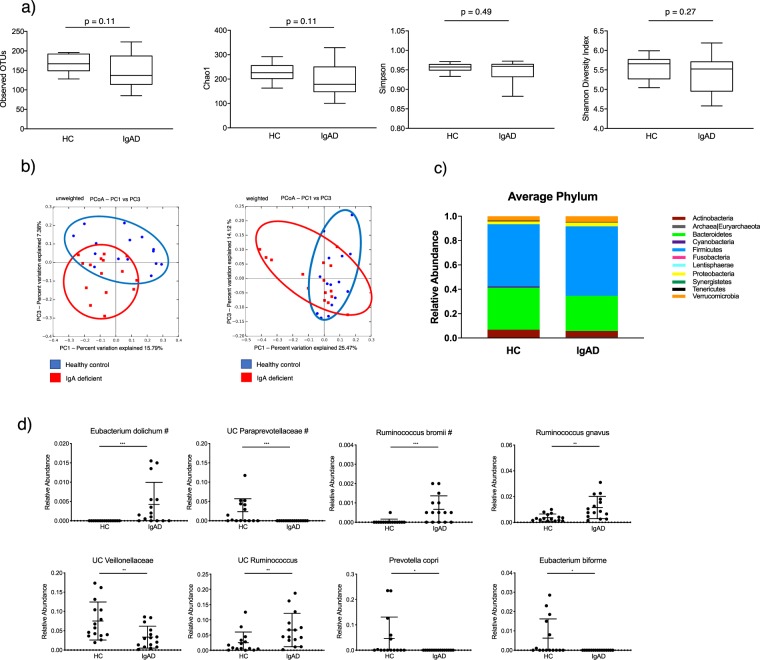
Humans with selective IgA deficiency harbor altered gut microbial communities. (**a**) Alpha diversity analyses of gut microbiota from sIgAd subjects and healthy controls (HC) as measured by observed species (OTUs), Chao1, Simpson and Shannon Diversity indices. (**b**) Principal Coordinate Analysis plots of unweighted and weighted UniFrac distances (**c**,**d**). Relative abundances of bacterial phyla (c) or species level taxa (**d**) exhibiting a significant difference between sIgAd and control subjects. *p < 0.01, **p < 0.001, ***p < 0.0001 by Mann-Whitney U test. ^#^indicates FDR < 0.05 using the Benjamini and Hochberg method using R console.

To assess the effect of IgA deficiency on gut microbial community composition, we performed 16S rRNA gene sequencing on fecal samples from fifteen sIgAd subjects and fifteen healthy controls. IgA has been suggested to support enhanced overall gut microbial diversity in mouse models^17^. Thus, we first evaluated microbiota diversity using a variety of metrics. We observed a trend toward decreased diversity in sIgAd subjects for multiple alpha diversity metrics (observed OTUs, Shannon Diversity Index and Chao1), but none of these differences reached statistical significance (p < 0.05) (Fig. 2a). As antibiotics are noted to influence the microbial diversity of the gut^18,19^, we also evaluated alpha diversity in the subset of sIgAd subjects who had not been exposed to antibiotics for at least 12 months prior to sample collection. We found that microbial diversity in these subjects was not significantly different from those who had taken an antibiotic between 1–12 months prior to sample collection (Supplemental Fig. 2). Additionally, using Spearman’s rank correlation coefficient, we found no correlation between time (in months) from previous antibiotic usage and Chao1 (Supplemental Fig. 3). Beta diversity analyses (Principal coordinate analysis based on weighted and unweighted Unifrac distances) revealed that microbial communities in sIgAd subjects occupied an overlapping but distinct cluster as compared to controls (Fig. 2b). We next used linear discriminant analysis effect size (LEfSe)^20^ to determine whether the relative abundances of specific bacterial taxa differed between sIgAd subjects and controls. At the phylum level, there was no statistically significant difference between the gut microbiota of sIgAd subjects and healthy controls (Fig. 2c, Supplemental Fig. 4). However, LEfSe analyses at all taxonomic levels revealed four taxa that were significantly more abundant (p < 0.01) in sIgAd subjects: *Eubacterium dolichum*, *Ruminococcus bromii*, *Ruminococcus gnavus*, and an unclassified (UC) *Ruminococcus* (Fig. 2d). Notably, both *Eubacterium dolichum* and *Ruminococcus bromii* were significantly increased in sIgAd subjects as compared to controls after correcting for false discovery rate (FDR) (Fig. 2d, Supplemental Table 1). In addition, two taxa were significantly less abundant in sIgAd subjects as compared to healthy controls (p < 0.01): UC *Veillonellaceae* and UC *Paraprevotellaceae*, and the latter of the two remained significant after correction for FDR (Fig. 2d,e, Supplemental Fig. 4). *Prevotella copri* and *Eubacterium biforme* fell just below the LEfSe significance value of p < 0.01 but were notably below the limit of detection in all IgA deficient subjects while remaining readily detectable in the majority of controls (Fig. 2d). Additional differentially abundant taxa were also apparent when we used a less-stringent significance cutoff in LEfSe (p < 0.05) (Supplemental Figs 5, 6). Taken together, these results suggest that the intestinal microbiota of subjects lacking secretory IgA is altered as compared to controls. Finally, we evaluated the relative abundance of specific taxa in sIgAd subjects who had not been exposed to antibiotics for at least 12 months prior to collection of the stool specimen to examine whether increased antibiotic exposure in sIgAd subjects may explain some of the differences described above. Other than UC Ruminococcus, all taxa that were significantly different in our prior comparisons of all sIgAd subjects versus controls were also significantly altered in sIgAd subjects with low rates of antibiotic exposure (Supplemental Fig. 7).

### Secretory IgA and compensatory secreted IgM display distinct bacterial binding patterns

**Figure 3:**
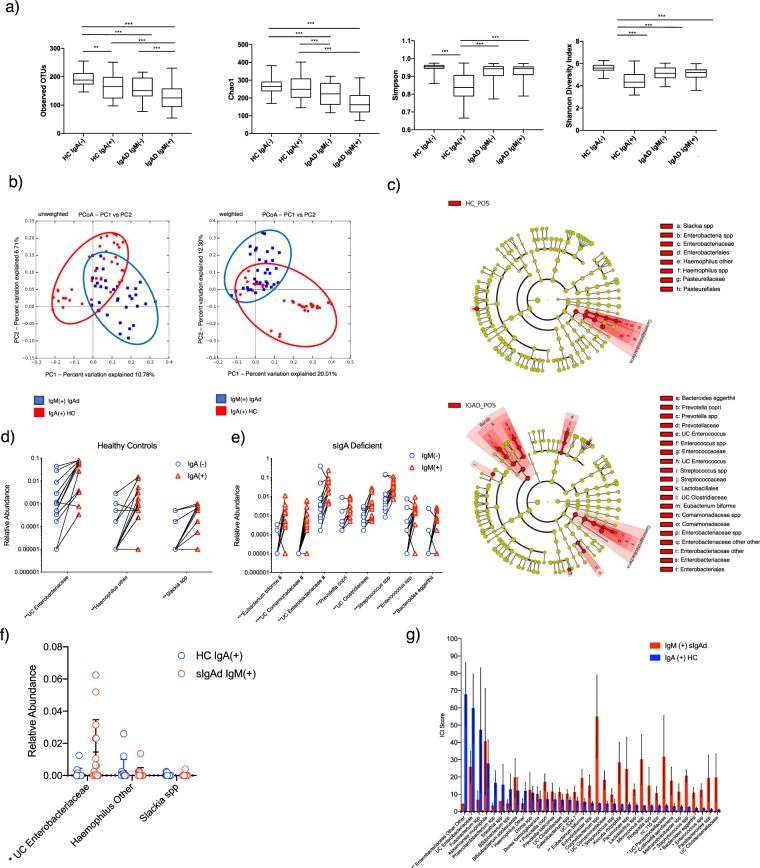
Secretory IgA in healthy controls and compensatory secreted IgM in sIgAd subjects display distinct patterns of bacterial coating. (**a**) Alpha diversity analysis (observed OTUs, Chao1, Simpson and Shannon Diversity indices) of antibody coated and non-coated fecal bacteria from healthy controls and sIgAd subjects. (**b**) Principal Coordinate Analyses of unweighted and weighted Unifrac distances for antibody coated and non-coated bacteria from healthy controls and sIgAd subjects. (**c**) Cladogram of linear discriminant analysis effect size (LEfSe) comparisons of secretory antibody coated versus non-coated bacteria from healthy controls (coated with IgA or non-coated) or sIgAd subjects (coated with IgM or non-coated) with p < 0.01. (**d,e**) Relative abundances of taxa that were significantly different between IgA-coated and non-coated fractions from control subjects (**d**) or between IgM coated and non-coated fractions in sIgAd subjects (**e**). (**f**) Relative abundances of bacterial taxa in healthy controls and sIgAd subjects that were previously demonstrated to be significantly more abundant in the IgA-coated fraction than non-coated fraction in healthy controls (**g**) Bar chart depicting mean IgA or IgM coating index scores (ICI scores) of taxa with mean ICI scores >10 in either sIgAd subjects or healthy controls. *p < 0.05, **p < 0.01, ***p < 0.001 by Mann Whitney U test. ^#^indicates FDR < 0.05 using the Benjamini and Hochberg method using R console. Bars indicate mean +/− SEM.

We previously developed a technology called IgA-Seq^21^. that combines bacterial cell sorting with 16S rRNA gene sequencing to determine the relative levels of IgA coating of the gut microbiota in a taxa-specific manner. We thus performed IgA-Seq or IgM-Seq on fecal bacteria from sIgAd subjects or healthy controls to identify which bacterial taxa were coated with either IgA (in controls) or IgM (in IgA-deficient subjects). To assess the overall distribution of Ig-coated versus non-coated taxa, we first evaluated the alpha diversity of the immunoglobulin coated and non-coated fractions in all groups (Fig. 3a). We observed that the non-coated fractions displayed a significantly higher species richness (as measured by observed OTUs) than the antibody-coated fractions in both IgA-deficient subjects and controls (Fig. 3a). Shannon and Simpson alpha diversity indices, which account for evenness as well as richness, were also significantly lower in the IgA-coated fraction versus non-coated fraction in healthy controls, suggesting that IgA coating in healthy individuals is targeted towards a limited subset of gut microbes (Fig. 3a). In contrast, in sIgAd subjects, IgM coated and non-coated fractions showed similar alpha diversity when quantified using the Shannon or Simpson indices (Fig. 3a). This pattern remained consistent when we restricted our analyses to subjects who had not been exposed to antibiotics for at least 12 months (Supplemental Fig. 8). This suggests that compensatory IgM targets a broad spectrum of bacterial taxa, while secretory IgA is more targeted towards specific species or strains. Beta diversity analyses (PCoA of weighted and unweighted Unifrac distances) revealed that IgA- versus IgM-coated microbial populations in healthy controls or sIgAd subjects clustered separately (Fig. 3b). In contrast, non-coated communities in sIgAd subjects versus controls showed significant overlap (Supplemental Fig. 9).

To identify which specific taxa were targeted by IgA or IgM, we next compared the taxonomic compositions of the Ig-coated versus non-coated fractions in IgA-deficient subjects and controls using LEfSe (Fig. 3c,d). These analyses revealed that *Haemophilus other*, *Slackia spp* and an UC *Enterobacteriaceae* were significantly IgA coated in healthy controls (enriched in the IgA coated fraction versus non-coated fraction; p < 0.01), though none of these differences were statistically significant after FDR correction (Fig. 3c,e, Supplementary Table 2). In contrast, a larger number of taxa were significantly coated with IgM (p < 0.01) in sIgAd subjects, including *Eubacterium biforme*, an UC *Comamonadaceae*, an UC *Enterobacteriaceae, Prevotella copri*, an UC *Clostridiaceae*, *Streptococcus spp*, an *Enterococcus genus*, and *Bacteroides eggerthii*; three of these (*Eubacterium biforme, an* UC *Comamonadaceae* and an UC *Enterobacteriaceae*) remained significantly different after FDR correction (Fig. 3c,e, Supplementary Table 3). We next evaluated the relative abundances of those taxa that were significantly more coated with antibody (either sIgA or sIgM) in sIgAd subjects versus controls. UC *Enterobacteriaceae*, which was heavily coated in both healthy controls and sIgAd subjects, was significantly more abundant (p < 0.05) in sIgAd subjects (Fig. 3f,g). This suggests that IgA coating acts to restrict expansion of this taxon and that IgM is unable to fully recapitulate this activity. In contrast, *Prevotella copri* and *Eubacterium biforme*, which were significantly coated with IgM in the sIgAd cohort, were more abundant in controls than in sIgAd subjects (Supplemental Fig. 10).

To quantify and directly compare the relative levels of secretory antibody coating between cohorts, we calculated an IgA or IgM coating index (ICI Score) for both populations by dividing the relative abundance of a given taxon in the coated fraction by the relative abundance in the non-coated fraction (ICI = relative abundance (IgA+ or IgM+)/relative abundance (IgA− or IgM−))^21^. We found that twenty nine taxa in the sIgAd cohort had average ICI scores greater than or equal to 10, while only ten taxa in the controls had similarly high ICI scores (Fig. 3g). Of the 39 taxa that had an average ICI > 10 in either cohort, nine taxa, UC *Paraprevotellaceae*, *Staphylococcus spp*, *Eubacterium biforme*, UC *Comamonadaceae*, *Collinsella aerofaciens*, *Prevotella copri*, *Enterococcus spp*, *Bacteroides eggerthii*, and a *Streptococcus spp*, were more highly coated by sIgM in the sIgAd cohort than by sIgA in the healthy control cohort (p < 0.05), though this significance was lost with application of Dunn’s multiple comparisons (Fig. 3g). In contrast, *Haemophilus* other and *UC Enterobacteriaceae* were more heavily coated with sIgA than compensatory IgM (p < 0.05), though again this significance was lost with application of Dunn’s multiple comparisons. When comparing the relative Ig coating of the eight taxa that demonstrated a significant difference in relative abundance in sIgAd versus controls (Fig. 2d), only *Prevotella copri* and *Eubacterium biforme* showed a trend towards higher coating with sIgM in the sIgAd subjects than sIgA in the healthy control cohort, but neither difference was significant after correction with Dunn’s multiple comparisons (Supplemental Fig. 11).

### Gut microbiota alterations in sIgAd subjects remain largely consistent over time

**Figure 4:**
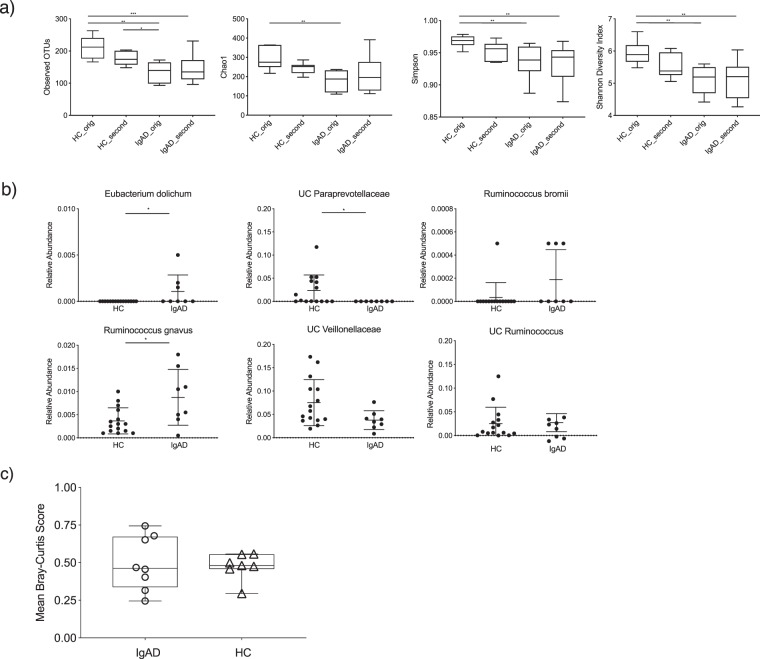
Alterations in gut microbiota composition in sIgAd subjects are durable across multiple timepoints. (**a**) Alpha diversity analyses of gut microbiota composition comparing initial and follow up stool samples from both healthy controls and sIgAd subjects as measured by observed OTU, Chao1, Simpson and Shannon Diversity Index. (**b**) Relative abundances of species level taxa from healthy controls and sIgAd subjects that had, in Fig. 2d, exhibited a significant difference between in relative abundance these two groups. (**c**) Bray-Curtis comparisons of initial and repeat samples from healthy control and IgA deficient subjects *p < 0.05, **p < 0.01, ***p < 0.001 by Mann Whitney U test.

Ecological models of community stability and resilience suggest that increased diversity is associated with increased overall stability and studies in mice suggest that secretory IgA can promote a more diverse microbial community^1,2^. To begin to characterize the stability of the fecal microbiota in sIgAd subjects, we collected repeat fecal samples from sIgAd subjects and controls 6–10 months after initial sample collection. Eight sIgAd subjects and seven healthy control subjects provided repeat samples. All subjects and controls reported no exposure to antibiotics at least one month prior to donation of the follow up sample. Furthermore, as seen in the baseline samples, time from last antibiotic exposure did not correlate with microbial alpha diversity (as measured by Chao1) in this cohort (Supplemental Fig. 12). We compared initial and repeat sampling of microbial communities from sIgAd subjects and controls using a variety of alpha diversity metrics (Fig. 4a). These analyses demonstrated that alpha diversity was largely consistent between initial and repeat microbiota collections for both cohorts. sIgAd subjects exhibited significantly lower alpha diversity than the initial healthy control sample (p < 0.05) for the majority of alpha diversity indices (Fig. 4a). Comparison of the taxonomic compositions of original and repeat samples by LEfSe revealed comparable microbiota stability in both sIgAd subjects and controls with no significant differences (p < 0.01) between initial and repeat samples in either group (Supplementary Tables 4, 5). Furthermore, three of the six taxa that were significantly altered in sIgAd subjects in our initial sample collection (Fig. 2d remained significantly different when comparing longitudinal samples from sIgAd and healthy controls (*Eubacterium dolichum*, UC *Paraprevotellaceae* and *Ruminococcus gnavus*)) (Fig. 4b). Also, UC *Paraprevotellaceae* remained below the level detection in sIgAd follow-up samples, and multiple longitudinal sIgAd samples contained detectable levels of *Eubacterium dolichum* and *Ruminococcus bromii*; these two taxa were detected only rarely or not at all in both initial and repeat samples from healthy controls (Figs 2d, 4b). These data suggest that alterations in the relative abundance of specific taxa in sIgAd subjects are durable over multiple months. Finally, we measured intra-individual microbiota stability over time by using the Bray-Curtis (BC) dissimilarity metric to compare changes in gut microbiota composition within individual subjects across time. Overall, we observed that sIgAd subjects and controls displayed similar mean BC dissimilarity over time, though IgA deficient subjects demonstrated greater intra-individual variability in dissimilarity between initial and repeat samples as compared to healthy controls (Fig. 4c).

## Discussion

Immunoglobulin A is best known for its role in protecting against infections at mucosal surfaces; however, it has recently become clear that IgA also plays a critical role in shaping the composition and function of the gut microbiota in mice. Selective IgA deficiency is the most common primary human immunodeficiency, which raises the question of whether IgA also plays a critical role in shaping gut microbial communities in humans. We addressed this question by examining gut microbiota composition and bacterial targeting by IgA and IgM in sIgAd subjects versus controls.

As IgA deficiency is largely asymptomatic, many subjects with sIgAd go undiagnosed. To obtain a collection of subjects that was broadly representative of the IgA-deficient community at large, we used the electronic medical record system at Yale to identify sIgAd subjects from the general New England population. These subjects’ IgA deficiencies were uncovered by means typical for this population—*e.g*., evaluation for recurrent infections or gastrointestinal disorders, or screening for Celiac disease. Thus, our recruitment strategy captured a population of sIgAd subjects that is representative of the sIgAd population seen in a variety of primary and subspecialty clinics.

We found that the gut microbiota in sIgAd subjects was altered as compared to healthy controls, with a trend towards an overall reduction in alpha diversity as well as defined changes in the presence and relative abundance of specific taxa. We also found that secretory IgM can at least partially compensate for the lack of IgA since IgM coated the gut microbiota of our IgA-deficient cohort at a level that was, on average, almost identical to the level of bacterial coating with IgA in controls. However, as compared to secretory IgA, compensatory IgM targeted a broader population of microbes; this suggests that compensatory IgM shows less specificity towards individual taxa or epitopes as compared to IgA. These data also imply that secretory IgA responses are, on average, more T cell-dependent and antigen-specific than compensatory IgM responses. Notably, sIgAd is thought to result from defective T-B interactions in at least a subset of IgA-deficient subjects^22^.

Initial results from our longitudinal sample collection effort demonstrate that alterations in gut microbiota composition in sIgAd subjects are consistent and durable over a time period of at least six months. However, these studies also suggest that the stability of the microbiota in IgA deficient subjects, while similar on average to controls, may display greater inter-individual variability. This highlights the possibility that select sIgAd subjects may experience more frequent deviations from a healthy microbial composition; such perturbations may play a role in the development or exacerbation of autoimmune or infectious diseases. Formally testing this possibility would require the establishment of a large, asymptomatic cohort of sIgAd subjects that would be monitored longitudinally for microbial perturbations and development of autoimmunity^13^. Ideally, this cohort would be free of antibiotic exposure both prior to and between sample collections; however, the significant association between sIgAd and increased infections would make this a challenging endeavor^23^.

IgA is classically conceptualized as a restrictive factor because of its ability to provide protection against mucosal pathogens^24^. Indeed, we noted that UC *Enterobacteriaceae*, which was highly coated by IgA in controls, showed increased relative abundance in sIgAd subjects as compared to healthy controls. Notably, mice with defective IgA responses also exhibit significant expansions in Proteobacteria^4^. Recent studies have revealed that IgA can also potentially enhance colonization by specific bacterial taxa either indirectly, through restriction of competitors, or by directly enhancing colonization of host-associated niches such as colonic crypts^25^. In line with these findings, we identified multiple taxa that were present in control subjects and nearly completely absent (or fell below the limit of detection) in sIgAd subjects. We also noted the absence of IgA sometimes had divergent effects on phylogenetically related species: *Eubacterium biforme* was observed exclusively in controls and was absent in sIgAd subjects, while *Eubacterium dolichum* was only seen in sIgAd subjects and not in controls. These data support the idea that IgA can have opposing effects on distinct members of the microbiota. Future studies will be necessary to understand what determines when IgA restricts versus promotes bacterial colonization or growth.

We used IgA- and IgM-Seq to determine taxa-specific patterns of targeting of the microbiota by IgA and compensatory IgM. We found that certain taxa, such as *UC Enterobacteriaceae* and *Akkermansia muciniphilia* were highly coated with both IgA and compensatory IgM (Fig. 3g). In contrast, select taxa were uniquely highly coated with IgA and showed lower levels of coating with compensatory IgM. This raises the possibility that distinct subsets of commensal bacteria may engage with the host in unique ways that lead to these divergent outcomes. The bacterial features or behaviors that define these distinctions will be an important subject of future research.

Two recent papers that were published while this report was under review also examined the impact of sIgAd on the human gut microbiota^15,17^. The results of these two studies and our own study share multiple similarities, but there are also notable differences. For example, while Fadlallah *et al*.^15^ found that the lack of IgA in the human gut did not markedly affect alpha diversity, our study and a recent study from Jorgensen *et al*.^26^ identified a significant decrease in alpha diversity in the sIgAd population. The specific alterations in gut microbiota composition in sIgAd subjects and the patterns of IgA and compensatory IgM coating that we observed also sometimes differed from the patterns observed by Fadlallah *et al*.^15^ and Jorgensen *et al*.^17^ All three studies found that some taxa from the *Ruminococcaceae* family were increased in sIgAd subjects, and our study and Fadlallah *et al*. observed both increased and decreased abundances of distinct taxa from the *Eubacterium* genus in sIgAd subjects. Furthermore, our study and Fadlallah *et al*. found that Proteobacteria were highly coated with IgA and displayed increased relative abundance in sIgAd subjects. These findings, coupled with prior mouse studies demonstrating IgA coating of Proteobacteria and expansion in the absence of effective IgA responses, suggest that IgA plays a critical and non-redundant role in restricting the expansion of Proteobacteria. Finally, we also observed seemingly contrary effects of IgA deficiency on the relative abundance of specific taxa (e.g., *Prevotella copri* and *Veillonellaceae*) as compared to Fadlallah *et al*.

Technical-, analytical-, and cohort-based differences may explain areas of divergence between these three studies. First, our study and the study by Jorgensen *et al*. used 16S rRNA gene sequencing to evaluate microbial community composition while Fadlallah *et al*. relied on a combination of shotgun metagenomics and 16S gene sequencing. In addition, Fadlallah *et al*. quantified alpha diversity based on the Shannon diversity index, while our study and the study by Jorgensen *et al*. used multiple alpha diversity metrics. Notably, we observed the strongest trends towards decreased alpha diversity in sIgAd subjects when evaluating diversity using OTUs or Chao1 and only observed a weak trend for the Shannon diversity index, while Jorgensen *et al*. observed a significant reduction in alpha diversity for all metrics including the Shannon diversity index. Differences in the clinical history of the subjects in the three studies may also explain some of the divergent findings: 52% of the sIgAd individuals in the Fadlallah *et al*.^15^ cohort presented with autoimmunity, whereas only 20% of our subjects had a known autoimmune condition. Also, the cohort in the Jorgensen *et al*. study was slightly older (41 years versus 32 years) than our cohort and had a higher incidence of recurrent infections (79% (11 of 14) versus 40% (6 of 15)). In addition, differences between European and American microbial communities, such as over or underrepresentation specific ‘enterotypes’ between continents, and varied diets between countries may also explain some of these seemingly discordant results^27^. Finally, because of the asymptomatic nature of IgA-deficiency, identifying subjects is often challenging and, thus, all three studies evaluated similarly small cohorts of subjects. Future studies employing larger cohorts that include subjects from multiple continents will be necessary to definitively identify universal features of microbial dysbiosis in humans with selective IgA-deficiency.

A final caveat for all studies of the microbiome in sIgAd is that sIgAd subjects on average experience more frequent antibiotic exposure than healthy controls due to their increased susceptibility to infection^23^. While antibiotic usage has been reported to have both short- and long-term effects on gut microbiota composition^18,19^, we did not observe any significant differences in alpha diversity between the total cohort of sIgAd subjects (selected based on no antibiotic use for at least four weeks before donation) and a subset of sIgAd subjects with no exposure to antibiotics for at least one year. This finding is similar to what was recently reported by Jorgensen *et al*.^26^ where they found no association between number of courses of antibiotics in the last year and gut microbiota alpha diversity. Furthermore, we found that specific microbial taxa that displayed altered abundance in our full cohort of sIgAd subjects were similarly altered in a subset of sIgAd subjects who had not been exposed to antibiotics for at least a year. Nonetheless, it is impossible to completely exclude the possibility that differences in antibiotic usage may contribute to the changes in gut microbiota composition in sIgAd subjects observed in this study and others.

In conclusion, our study suggests that IgA plays a critical and non-redundant role in shaping the composition of the gut microbiota in humans and that IgM can partially, but not completely, compensate for the absence of IgA. Thus, while the relatively high rate of asymptomatic sIgAd has been used to argue against the importance of IgA, our studies highlight the critical role of specialized secretory antibody isotypes in the maintenance of host-microbiota symbiosis.

## Methods

### Sample collection

Human study protocols were approved by the Institutional Review Board (HIC # 1607018104) of the Yale School of Medicine, New Haven, CT. Informed consent was obtained from all participants and/or their legal guardians and all methods were performed in accordance with relevant guidelines and regulations. IgA deficient subjects were identified via the EPIC electronic medical record system and all subjects resided in the state of Connecticut. Demographics, medical history and other clinical variables were collected following enrollment. Healthy subjects were recruited via advertisements on the Yale medical campus and in the New Haven Public Library. All fecal samples in this study were collected at home and stored on ice packs at −20 °C before either overnight shipment or direct drop-off the day following collection in the insulated container provided. Samples were then stored at −80 °C until use.

### Measurement of serum immunoglobulins

Whole blood was drawn by venipuncture into tubes without anticoagulant (BD, USA). Blood was allowed to clot for 30 minutes, centrifuged at 10,000 × g for 10 minutes, and serum supernatant was collected. Serum was probed for IgA and IgM via ELISA (coating Sigma SAB3701393, Detection of IgA1 Southern Biotech 9130-08, IgA2 Southern Biotech 9140-08, IgM Southern Biotech 9020-08). The lower limit of sensitivity for measurement of intestinal immunoglobulins is 0.3 ug/mL for IgA and 0.2 ug/mL for IgM.

### Bacterial flow cytometry, IgA-Seq and IgM-Seq analyses

Fecal flow cytometry and IgA-Seq were performed essentially as previously described except that Ig-coated bacteria were isolated using MACS beads and a custom-built 96 well magnetic separator (https://www.kjmagnetics.com/proddetail.asp?prod=D28-N52) followed by negative selection using MACS multi 96 columns^21^. Bacteria were stained with either PE-conjugated Anti-Human IgA (1:10; Miltenyi Biotec clone IS11-8E10) or FITC- or PE- conjugated Anti-Human IgM (1:50 JI 109-096-008). IgM-Seq samples were processed identically to IgA-Seq except that PE-anti-IgM was used in place of PE-anti-IgA. The estimated bacterial numbers in all samples post-separation exceeded 1 × 10^7^ bacteria per fraction^21^.

### 16S ribosomal RNA gene sequencing and microbial community composition analysis

The V4 region of the 16S ribosomal RNA gene was sequenced on a MiSeq (2 × 250; Illumina) as described previously^21,28,29^. Microbial diversity analyses were performed using the Quantitative Insights into Microbial Ecology (QIIME version 1.9) analysis suite. Reads were assembled, demultiplexed and quality filtered with a Q-score cutoff of 30. Open-reference OTU picking against the Greengenes reference database was used to cluster into Operational Taxonomic Units (OTUs) at 97% identity. The Ribosomal Database Project classifier (RDP) and the May 2016 Greengenes taxonomy were used to assign taxonomy to representative OTUs^30^. OTUs of less than 0.01% relative abundance and contaminating OTUs found in water-only controls were removed from OTU tables. QIIME was used for all microbial ecology analyses^31^ and Linear Discriminant Analysis Effect Size (LEfSe)^20^ was used to compare taxonomic abundance between groups. For characteristic analyses and diversity indices between groups, differences were estimated by the Mann Whitney U-test with Dunn’s multiple comparison’s in Prism GraphPad Software. P values were corrected to control for the false discovery rate (FDR) using the Benjamini-Hochberg method in R console.

### Study approvals

The study protocol was approved by the Yale University Institutional Review Board (HIC#1607018104). Written informed consent was received from participants prior to inclusion in the study.

## Supplementary information

Supplementary Title with Legends and Figures

Supplementary Table 1

Supplementary Table 2

Supplementary Table 3

Supplementary Table 4

Supplementary Table 5

## Data Availability

The raw sequence reads for all studied described above have been deposited in the NCBI Sequence Read Archive (SRA) Database in FASTA format. The accession numbers for the BioProject associated with this paper are PRJNA558911 and PRJNA558378.
