# Supplementary material for: IgA-deficient humans exhibit gut microbiota dysbiosis despite secretion of compensatory IgM
Source: Sci Rep. 2019 Sep 19;9:13574. doi: 10.1038/s41598-019-49923-2 (PMC6753154; doi:10.1038/s41598-019-49923-2)

SUPPLEMENTARY FIGURES FOR:

Title: IgA-deficient humans exhibit gut microbiota dysbiosis despite secretion of compensatory IgM

*Jason R Catanzaro[1], Juliet D Strauss[2,3], Agata Bielecka[2], Anthony F Porto[4], Francis M Lobo[5], Andrea Urban[6], Whitman B Schofield[2,3], Noah W Palm[2]

Author Affiliations

1. Section of Pulmonology, Allergy, Immunology, and Sleep Medicine, Department of Pediatrics, Yale School of Medicine, New Haven, CT, USA
2. Department of Immunobiology, Yale School of Medicine, New Haven, CT, USA
3. Artizan Biosciences, New Haven, CT, USA
4. Section of Pediatric Gastroenterology, Department of Pediatrics, Yale School of Medicine, New Haven, CT, USA
5. Section of Rheumatology, Allergy and Clinical Immunology, Department of Internal Medicine, Yale School of Medicine, New Haven, CT, USA
6. Section of Pediatric Endocrinology, Department of Pediatrics, Yale School of Medicine, New Haven, CT, USA

LEGEND:

Supplemental Figure 1 | Representative flow cytometric analysis of IgA coated bacteria in a healthy subject and IgM coated bacteria in an IgA deficient subject.

Supplemental Figure 2 | Alpha Diversity analyses in healthy controls (HC), sIgAd subjects (all, >12m: no antibiotics in >12 months, <12m: treated with antibiotics within 12 months) as measured by observed species (OTU), Chao1 and Shannon Diversity Index

Supplemental Figure 3 | Scatterplot comparison time from last antibiotic in months and Chao1. r: Spearman rank correlation coefficient.

Supplemental Figure 4 | Bar plots depicting relative abundances of phyla from fifteen sIgAd subjects and healthy controls.

Supplemental Figure 5 | Cladogram generated from linear discriminant effect size (LEfSe) comparisons between healthy controls and sIgAd subjects with $p < 0.05$.

Supplemental Figure 6 | Linear discriminant analysis (LDA) scores for differentially abundant taxa enriched in healthy controls versus sIgAd subjects with $p < 0.05$.

Supplemental Figure 7 | Relative abundances of species level taxa in sIgAd subjects who had not received an antibiotic in the previous twelve months versus healthy controls. *p<0.05, **p<0.01

Supplemental Figure 8 | Alpha Diversity analyses (observed species (OTU), Chao1 and Shannon Diversity Index) of antibody coated and non-coated fecal bacteria from healthy controls (HC) and sIgAd subjects (all, >12m: no antibiotics in >12 months, <12m: did receive antibiotics within 12 months). *p<0.05, **p<0.01, ***p<0.001 by Mann Whitney U test.

Supplemental Figure 9 | Principal coordinate analyses of unweighted and weighted UniFrac distances for uncoated taxa from healthy controls and sIgAd subjects.

Supplemental Figure 10 | Relative abundances of bacterial taxa in sIgAd subjects and healthy control subjects that were previously demonstrated to be significantly more abundant in the IgM coated fraction than non-coated in sIgAd subjects *p<0.05, **p<0.01, ***p<0.001 by Mann Whitney U test.

Supplemental Figure 11 | ICI scores for the 8 taxa that were demonstrated significantly different relative abundances between sIgAd and healthy controls. *p<0.05 by Mann Whitney U test.

Supplemental Figure 12 | Scatterplot comparison of time from last antibiotic in months and Chao1 for sIgAd subjects who provided a second sample 6-10 months after initial sample collection. r: Spearman rank correlation coefficient.

Supplemental Table 1 | Relative abundances of taxa for each healthy control or IgA deficient subject with p values from Mann Whitney U test and q values from the false discovery rate (FDR) using the Benjamini and Hochberg method

Supplemental Table 2 | Relative abundances of taxa for each healthy control either IgA coated (HCPOS) or noncoated (HCNEG) with p values from Mann Whitney U test and q values from the false discovery rate (FDR) using the Benjamini and Hochberg method

Supplemental Table 3 | Relative abundances of taxa for each sIgAd subject either IgM coated (IGAD_POS) or noncoated (IGAD_NEG) with p values from Mann Whitney U test and q values from the false discovery rate (FDR) using the Benjamini and Hochberg method

Supplemental Table 4 | Relative abundances of taxa for each subject either HC – initial sample (HC_orig) or HC – follow up sample (HC_second) with p values from Mann Whitney U test and q values from the false discovery rate (FDR) using the Benjamini and Hochberg method

Supplemental Table 5 | Relative abundances of taxa for each subject either sIgAd – initial sample (sIgAd_orig) or sIgAd – follow up sample (sIgAd_second) with p values from Mann Whitney U test and q values from the false discovery rate (FDR) using the Benjamini and Hochberg method

S1)

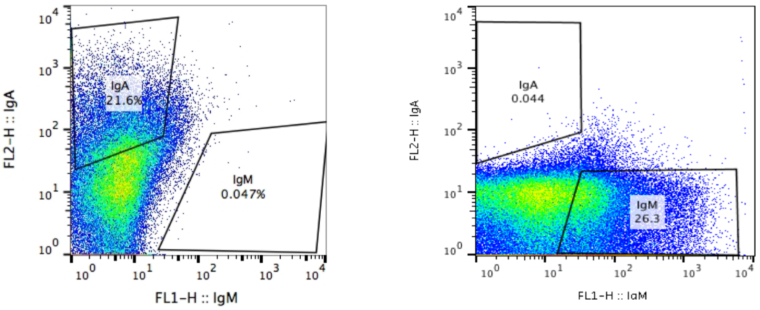

S2)

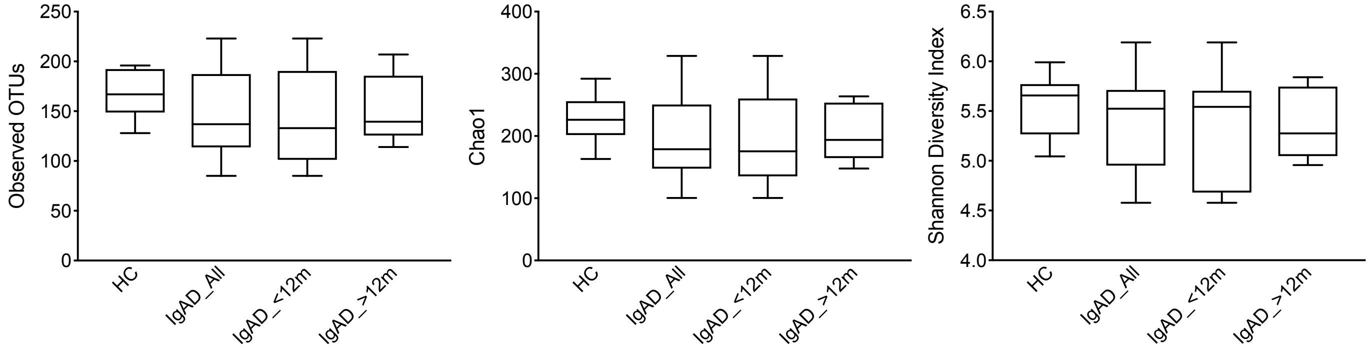

S3)

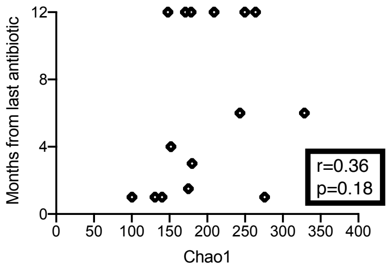

S4)

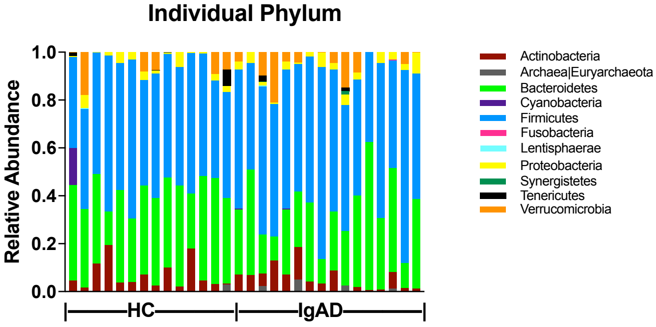

Healthy control and sIgAD

S5)

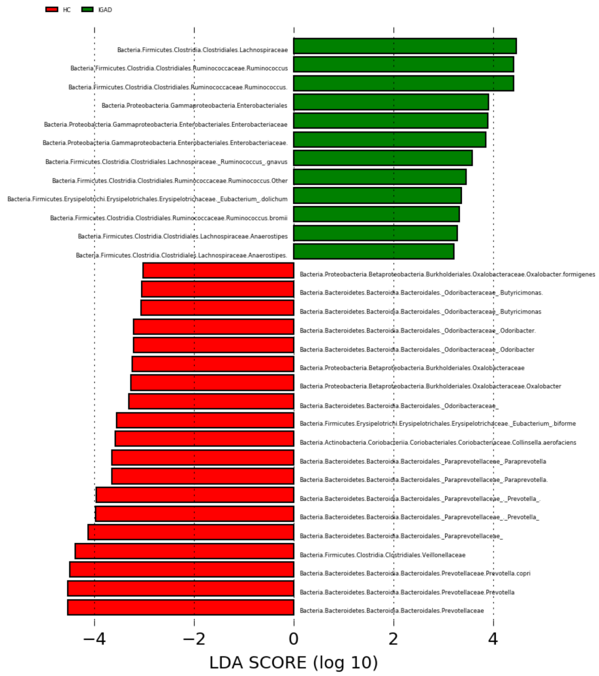

S6)

Healthy control and sIgAD

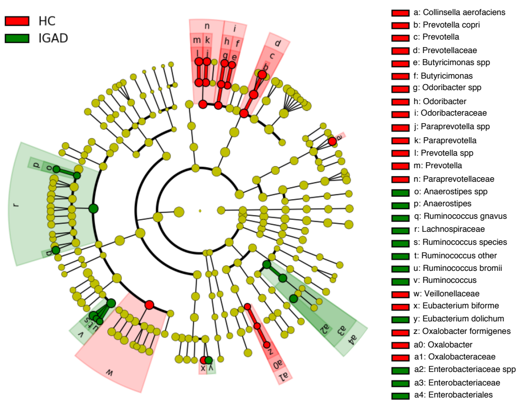

S7)

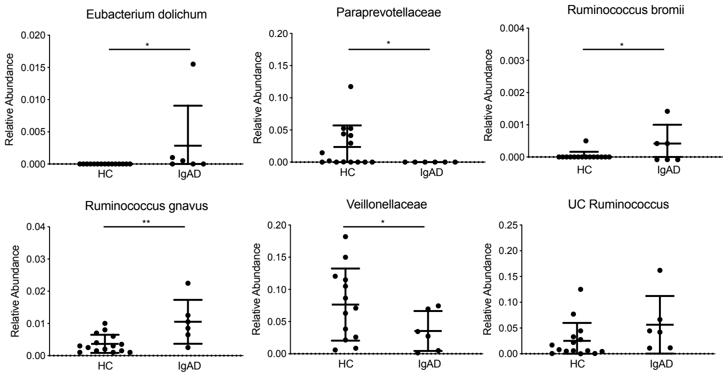

S8)

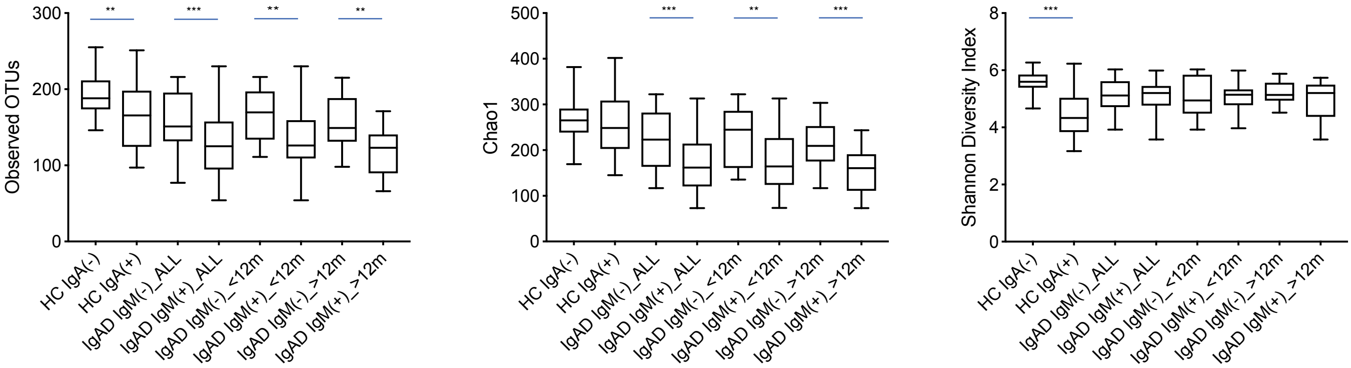

S9)

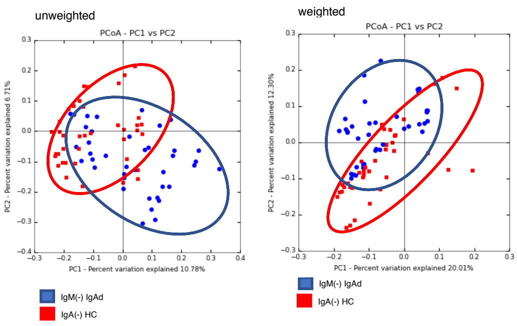

S10)

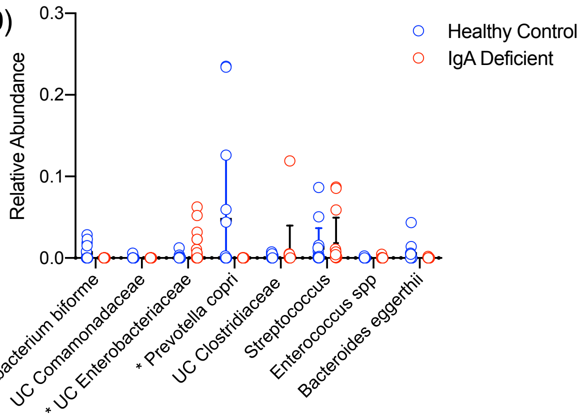

S11)

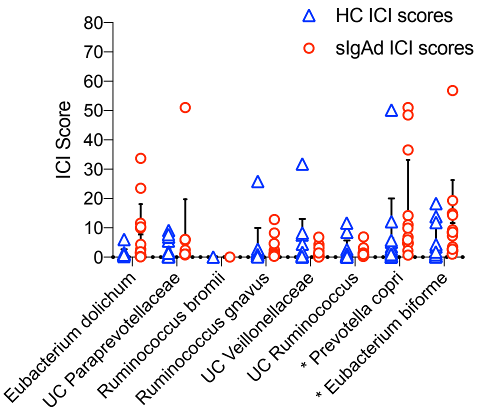

S12)

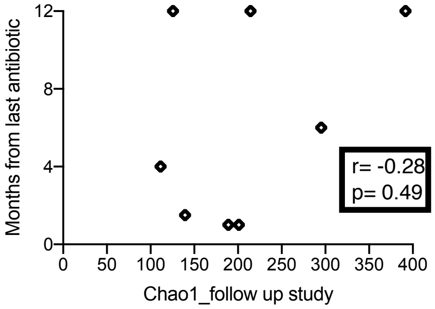

Supplement: Supplementary file 1 — Supplementary Title with Legends and Figures [file 41598_2019_49923_MOESM1_ESM.pdf]
